# New insight into the redox activity of niclosamide, an anthelmintic drug, and some of its metabolites. A new perspective on toxicity and detoxification

**DOI:** 10.1039/d6ra02391f

**Published:** 2026-07-06

**Authors:** Davood Nematollahi, Hadis Khazaei-Rahmati

**Affiliations:** a Faculty of Chemistry and Petroleum Sciences, Bu-Ali Sina University Hamedan 65178-38683 Iran nemat@basu.ac.ir; b Plant Chemistry Research Center, Bu-Ali Sina University Hamedan Iran

## Abstract

Redox activity of niclosamide (NIC) and its metabolites 2-chloro-4-nitroaniline (2CN) and 5-chlorosalicylic acid (5CA) has been comprehensively investigated over a wide pH range. In this work, to gain a better understanding of the electrochemical activity of NIC, its oxidation and reduction have been investigated independently, and the results are compared with those of 2CN, 5CA, and 4-nitroaniline (4NA) (as a model compound). The results show that both oxidation and reduction pathways of NIC are more complex than those of 2CN, 5CA, and 4NA. While the oxidation of the cathodically generated 2-chloro-*p*-phenylenediamine (2CA) and *p*-phenylenediamine (PPD) produces relative quinonediimines, the oxidation of reduced NIC (NIA) leads to the corresponding chloroniumquinoneimine (NCC). The strong intramolecular electrostatic interaction between the carbonyl oxygen and the positively charged chlorine atom has stabilized the NCC structure. The lack of this interaction during the oxidation of 2CA makes the formed chloroniumquinoneimine (2CC) unstable and converts it to the corresponding quinonediimine. Our results also show that the generated NCC can react with nucleic acids or proteins; however, glutathione can rapidly react with NCC. The predominant reaction of glutathione with NCC and quinonediamines is catalytic. We have also reported for the first time that the nitro group in NIC, 2CN, and 4NA molecules undergoes catalytic reduction in the presence of oxidized glutathione. The results of this study provide new insights into the redox chemistry of NIC that can be used in the synthesis of next-generation drugs with improved pharmacological and physicochemical properties.

## Abstract 2

Redox activity of niclosamide (NIC) and its metabolites 2-chloro-4-nitroaniline (2CN) and 5-chlorosalicylic acid (5CA) has been comprehensively investigated over a wide pH range.

## Graphical abstract

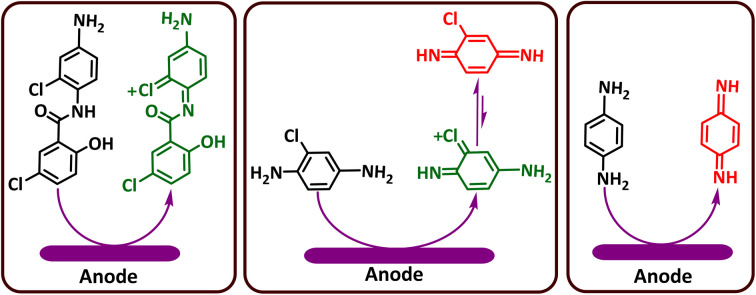

## Introduction

Studying the chemical and biochemical properties of drug metabolites helps to better understand the mechanism of drug action, leading to the development of new drugs with greater efficacy and fewer side effects. In other words, understanding the role of drug metabolites is a critical step in the development of new drugs. It provides deep insight into the potential toxicity caused by the metabolites formed.^1^ Most drug metabolism occurs in the liver and involves biotransformations that aim to inactivate the drug and enhance its excretion by increasing the polarity of the compound.^2^ For this purpose, the drug is first converted to polar metabolites in the presence of CYP-450 enzyme isoforms by participating in reactions such as oxidation, reduction, or hydrolysis.^3^ The metabolite then reacts with compounds such as glucuronic acid, glutathione, acetyl-CoA, S-adenosylmethionine, glycine, water, or phosphoadenosyl phosphosulfate^4,5^ to form products with greater water solubility and can therefore be excreted *via* bile or urine.^6,7^

From the above, it can be concluded that understanding the role and properties of metabolites is essential for discovering the fundamental mechanisms of health and disease. However, the biotransformation of drugs and toxicological studies are costly and time-consuming.^8^ Since electrochemical oxidation often parallels cytochrome P450-catalyzed oxidation in liver microsomes, electrochemical methods can be used for synthesis, characterization of redox activities, and toxicological studies of metabolites. Unlike drug biotransformation, the synthesis of metabolites by chemical and/or electrochemical methods and their toxicological studies by electrochemical methods are inexpensive and rapid.^8^

In this study, niclosamide (NIC), an anthelmintic drug, is the target of our research. To demonstrate the increasing importance of this drug in the treatment of various diseases, a quick search on Google Scholar reveals over 1700 results for this drug in 2025 alone. This drug, currently used to treat parasitic infections, has attracted the attention of many researchers due to its potential therapeutic application in diabetes, Parkinson's disease, various cancers, as well as viral and microbial infections.^9,10^ Despite the discovery of NIC in the early 1950s, its mechanism of action is still not fully understood. Early studies have shown that niclosamide's activity is due to the uncoupling of oxidative phosphorylation.^10^

Despite the high pharmacological potential of this drug, its clinical efficacy is affected by its toxicity. For example, this drug has been reported to have high acute toxicity to fish and non-target aquatic organisms.^11,12^ Also, although NIC has been widely used as an anticancer agent and has shown significant cytotoxic/cytostatic effects *in vitro*, its toxicity has raised concerns.^13^ It has also been shown that NIC can cause hepatotoxicity and immunogenicity in *M. piceus*.^14^

In our recent research on the synthesis of some NIC derivatives, we reported that after reduction of the nitro group to the amine group, the reduced drug (NIA) is oxidized at the anode surface.^15^ The chemical similarity of the oxidized-NIA to the *para*-quinoneimine produced from the oxidation of acetaminophen has raised the hypothesis that, like acetaminophen,^16,17^ this intermediate may be the cause of NIC hepatotoxicity. In this work, we synthesized some metabolites of NIC and comprehensively investigated their redox activity under different conditions and compared them with the data of NIC. We also investigated the reaction of oxidized-NIA and quinonediimines with glutathione to elucidate their reaction mechanism. Our results suggest that oxidized-NIA and quinonedimines can participate in a catalytic reaction with glutathione. This reaction confirms that these oxidized species are also capable of reacting with proteins or nucleic acids and causing liver damage.

## Results and discussion

### Electrochemical reduction studies

**Scheme 1:**
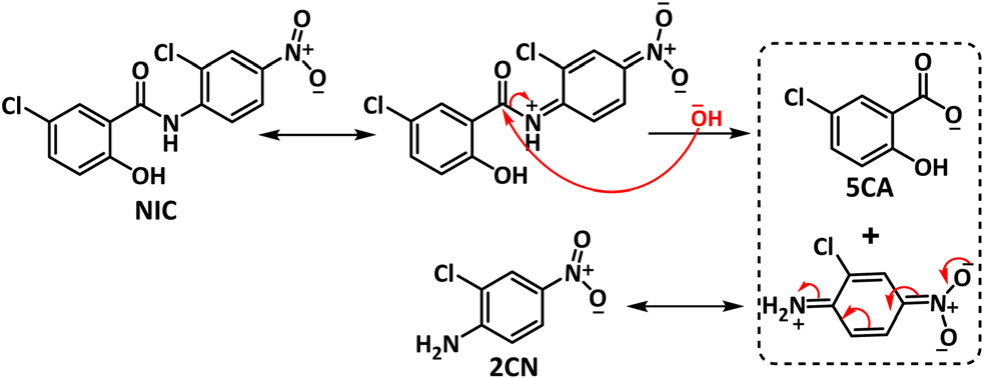
Possible proposed mechanism for the alkaline hydrolysis of NIC.

Genotoxic studies in humans and rodents indicate that NIC is absorbed from the gastrointestinal tract and converted to the mutagenic metabolites 2-chloro-4-nitroaniline and 5-chlorosalicylic acid.^18^ In this work, these metabolites, 2-chloro-4-nitroaniline (2CN) and 5-chlorosalicylic acid (5CA), were synthesized by alkaline hydrolysis of NIC,^19^ and their redox activity was comprehensively investigated and compared with NIC as well as 4-nitroaniline (4NA) as a model compound. The possible proposed mechanism for the alkaline hydrolysis of NIC is shown in Scheme 1.

**Fig. 1:**
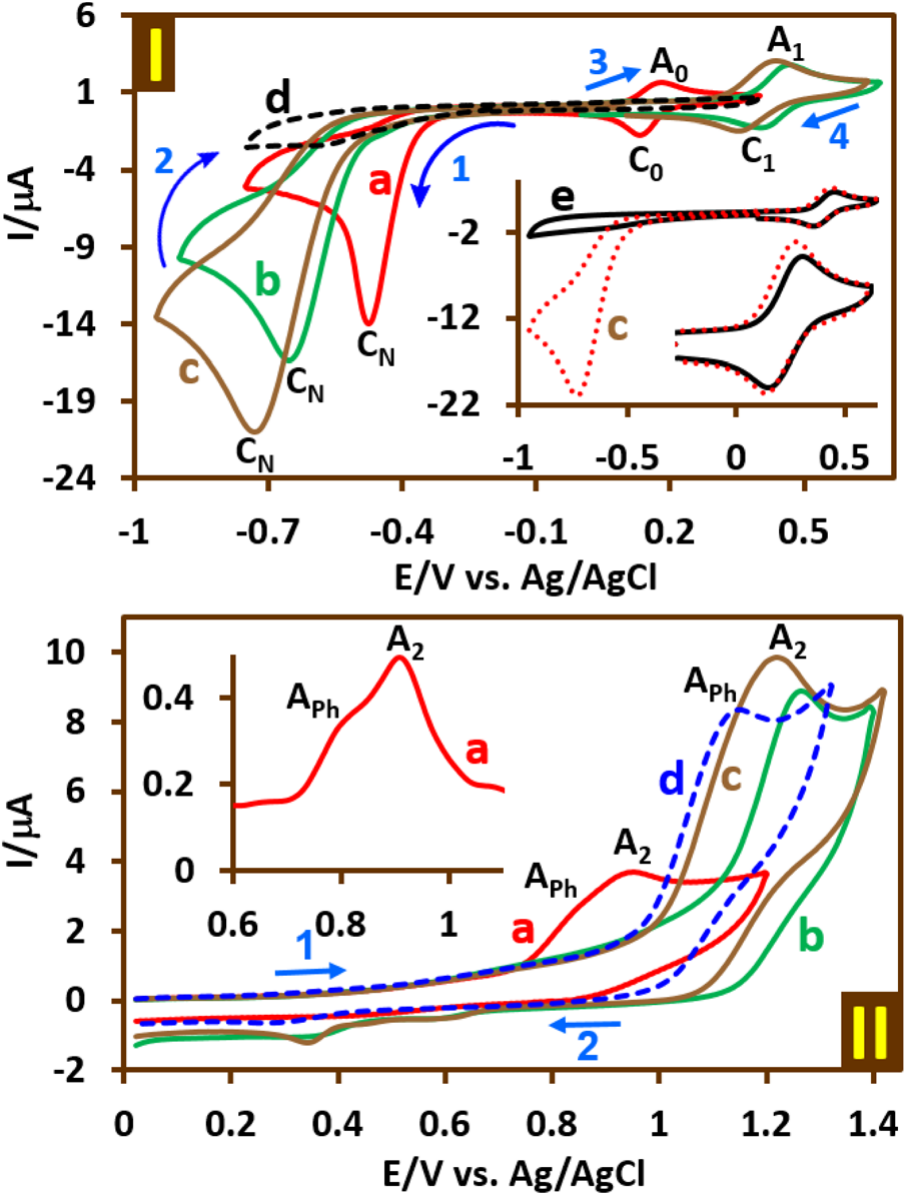
Part I: cyclic voltammograms of (a) NIC (1.0 mM), (b) 2CN (1.0 mM), (c) 4NA (1.0 mM), and (d) 5CA (1.0 mM). Inset: (e) cyclic voltammogram of PPD (0.3 mM). Part II: cyclic voltammograms of (a) NIC (1.0 mM), (b) 2CN (1.0 mM), (c) 4NA (1.0 mM), and (d) 5CA (1.0 mM), when only anodic scanning has been performed. Inset: differential pulse voltamogram of NIC (0.5 mM). Working electrode: glassy carbon electrode. Scan rate: 100 mV s^−1^. Part I, solvent: water (phosphate buffer, pH 2, *c* = 0.2 M)/ethanol (50/50 v/v). Part II, solvent: water (phosphate buffer, pH 2, *c* = 0.2 M)/ethanol (80/20 v/v). All voltammograms were recorded at room temperature. Arrows indicate potential sweep direction.

Fig. 1, part I, shows the cyclic voltammograms of NIC, 2CN, 4NA, and 5CA at pH 2. To record these voltammograms, the potential scan was performed in three steps. First, the potential was scanned in the cathodic direction. The result of this potential scan is the appearance of the C_N_ peak. In the next step, the potential scan direction was changed towards the anode, which resulted in the appearance of the peak A_0_ for NIC (A_1_ for 2CN and 4NA), and in the last step, the cathodic scan was performed again, which resulted in the appearance of the cathodic peak C_0_ for NIC (C_1_ for 2CN and 4NA) associated with the peak A_0_.

**Scheme 2:**
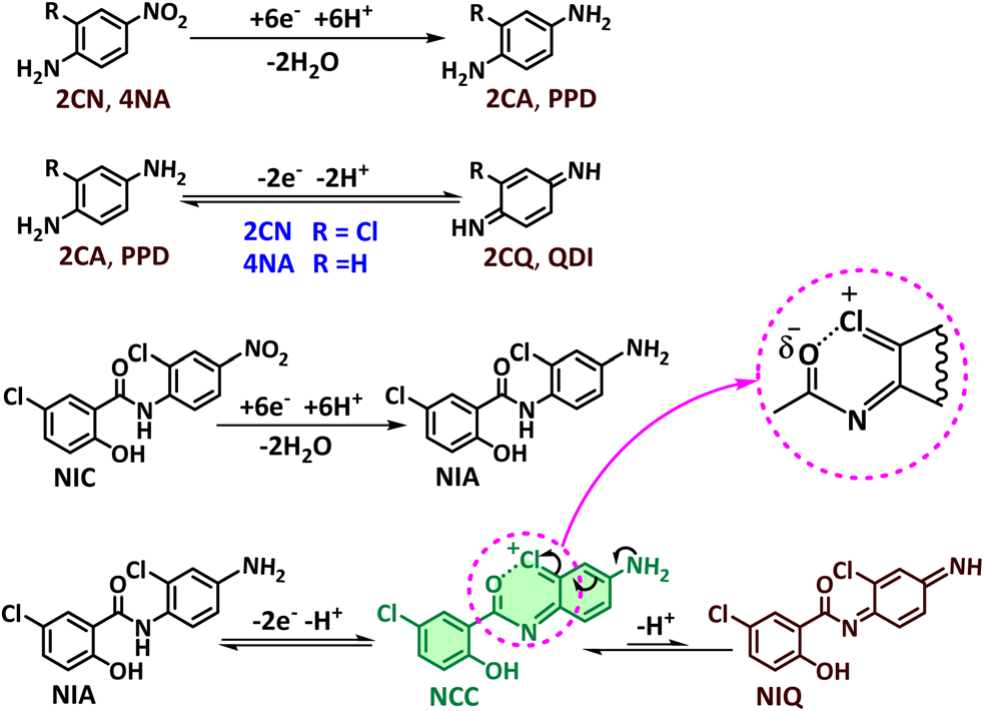
Possible proposed mechanism for the redox activity of 2CN, 4NA, and NIA.

It should be noted that in all three compounds, the presence of peaks A_0_ (or A_1_) and C_0_ (or C_1_) is dependent on the C_N_ peak, so that if a cathodic scan is not performed initially, peaks A_0_ (or A_1_) and C_0_ (or C_1_) will no longer be seen. According to previously published results on the redox behavior of NIC,^15^ the peak C_N_ is related to the reduction of the nitro group present in NIC, 2CN, and 4NA molecules to the amine group (formation of NIA, 2-chloro-*p*-phenylenediamine, 2CA, and *p*-phenylenediamine, PPD) (Scheme 2). The formation of 2CA and PPD can be confirmed by performing exhaustive electrolysis and following the reaction products by TLC. To more confirm the above statement, in the inset figure, the cyclic voltammogram of PPD recorded with the same potential scan program is compared with the cyclic voltammogram of 4NA. The coincidence of peaks A_1_ and C_1_ in these two compounds (4NA and PPD) clearly confirms that 4NA is converted to PPD after reduction of the nitro group (Fig. 1, part I, Inset). The potential of peak C_N_ (*E*^p^_N_) is −0.47, −0.67, and −0.73 V *vs.* Ag/AgCl, for NIC, 2CN, and 4NA molecules, respectively. Comparing the *E*^p^_N_ values in the 2CN and 4NA molecules shows that the nitro group in the 2CN molecule is reduced slightly more easily. The electron-withdrawing chlorine atom in 2CN appears to reduce the nitro group of 2CN slightly more easily compared to 4NA. On the other hand, the presence of a carbonyl group with a strong electron-withdrawing nature attached to the nitrogen atom in NIC makes the reduction of the nitro group in NIC easier than in 2CN. It should be noted that in this figure, the cyclic voltammogram *d* corresponds to 5CA and, as expected, lacks the peak related to the reduction of the nitro group. Peak A_0_ (or A_1_) corresponds to the oxidation of NIA, 2CA, or PPD, and peak C_0_ (or C_1_) is its counterpart. Comparison of the cyclic voltammograms of 4NA with PPD shows that peak A_1_ corresponds to the two-electron oxidation of PPD to the corresponding quinonediimine (QDI) and peak C_1_ is its counterpoint (Scheme 2).^20–23^ The peak A_0_ (or A_1_) potentials for the oxidation of NIA, 2CA, and PPD are 0.18, 0.47, and 0.43 V *vs.* Ag/AgCl, respectively. The more difficult oxidation of 2CA (*E*^p^_A1_ = 0.47 V) compared to PPD (*E*^p^_A1_ = 0.43 V) was predictable due to the presence of the chlorine atom with its electron-withdrawing nature in the structure of 2CA.

The important point in comparing these potentials is that NIA is much more readily oxidized than 2CA and PPD. Our results show that NIA oxidation occurs 250 mV lower than that of 2CA and PPD. It appears that the two-electron oxidation of NIC under current laboratory conditions takes a different route and leads to the corresponding chloroniumquinoneimine (NCC) due to the presence of a chlorine atom and an amide group in the molecule.^24^ Although NCC is a more unstable compound and seems to be easily converted to NIQ (Scheme 2), the presence of a strong intramolecular electrostatic interaction between carbonyl oxygen and positive chlorine atom has stabilized the NCC structure, such that under the current experimental conditions, no trace of NIQ peak is observed in the voltammograms. At the end of this section, it is important to note that under these conditions, no cathodic or anodic peaks are observed in the cyclic voltammogram of 5CA (Fig. 1, part I, CVd).

### Electrochemical oxidation studies

**Scheme 3:**
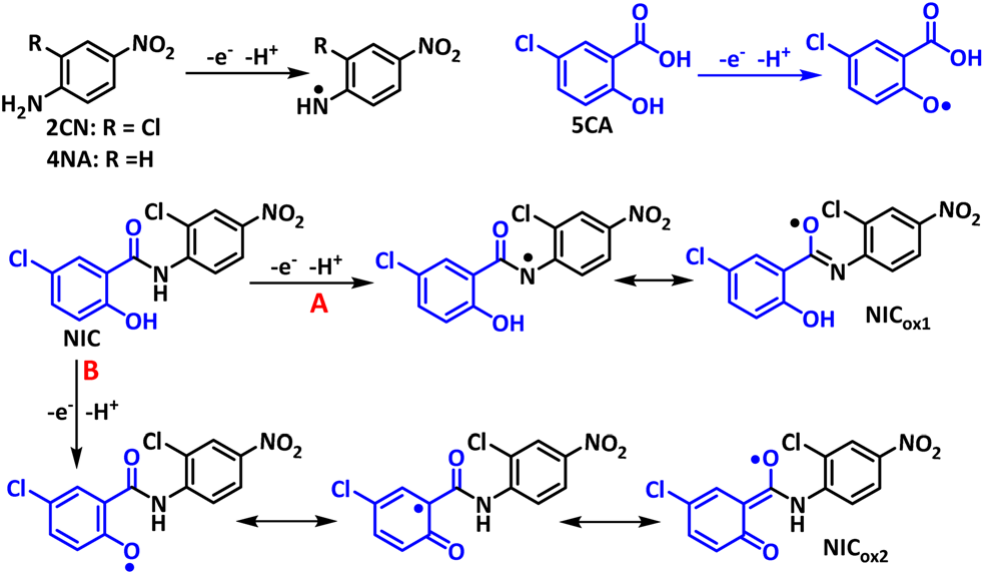
Possible proposed mechanism for the first electron transfer step in the oxidation of NIC, 2CN, 4NA, and 5CA.

Here, we also investigated the oxidation of NIC and its resulting metabolites (Fig. 1, part II). As can be seen, the oxidation of all studied compounds occurs through an irreversible process. Now, if we consider the simplest molecule, 4NA, we conclude that the A_2_ peak is related to the removal of one electron and one proton from the amine group and the formation of the corresponding radical^25^ (Scheme 3). Under these experimental conditions, the oxidation peak of 4NA (*E*^p^_A2_) appears at 1.22 V. Under the same conditions, the oxidation peak of 2CN appears at 1.27 V. As mentioned earlier, the presence of a chlorine atom in the 2CN structure is the reason for a more positive oxidation peak potential. 5CA also has an irreversible oxidation peak (A_Ph_) at a potential of 1.15 V, which is related to the single-electron oxidation of 5CA and the formation of the phenoxy radical (Scheme 3). What happens after the electron transfer step in the case of this compound is similar to what has been reported in the oxidation of phenolic compounds^26,27^ or salicylic acid.^28–31^ In the case of NIC itself, the voltammogram is a bit more complicated. There are two oxidation pathways for this molecule. The first (pathway A) involves the removal of an electron from the nitrogen atom of the amide group and the formation of NIC_ox1_. The second pathway involves the removal of an electron and a proton from the oxygen atom of the hydroxyl group and the formation of NIC_ox2_ (Scheme 3). Resonance stability makes the radicals formed from NIC oxidation more stable than 2CN and 4NA radicals, thereby facilitating NIC oxidation. Furthermore, the existence of two oxidation pathways for NIC and the formation of two different compounds, NIC_ox1_ and NIC_ox2_, is the reason for the presence of two anodic peaks (A_2_ and A_Ph_) in the voltammogram of NIC (Fig. 1, part II, inset).

**Fig. 2:**
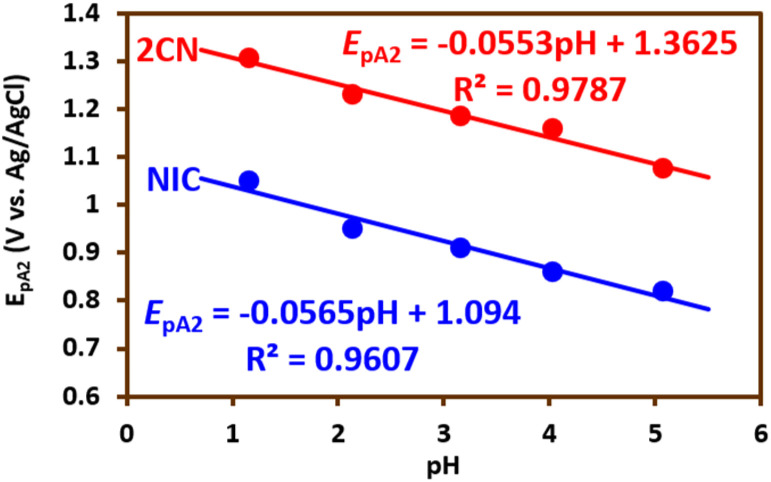
*E* ^p^_A2_-pH diagram for NIC and 2CN.

Another feature of the oxidation of NIC, 2CN, 4NA, and 5CA is the dependence of *E*^p^_A2_ on pH. The results of voltammetric studies show that with increasing pH, *E*^p^_A2_ shifts towards less positive potentials. The *E*^p^_A2_-pH diagram for NIC and 2CN are shown in Fig. 2. The slopes of the lines for the oxidation of NIC and 2CN are 56.5 and 55.3 mV per pH, respectively. In this type of diagram, the slope of the lines is “59.2 × *m*/*n*” mV per pH, where *m* is the number of protons and *n* is the number of electrons participating in the electrochemical process. The slopes of the NIC and 2CN lines are close to 59.2, indicating the participation of one electron and one proton (*m* = *n* = 1) in the oxidation of these compounds.

### The effects of pH

**Fig. 3:**
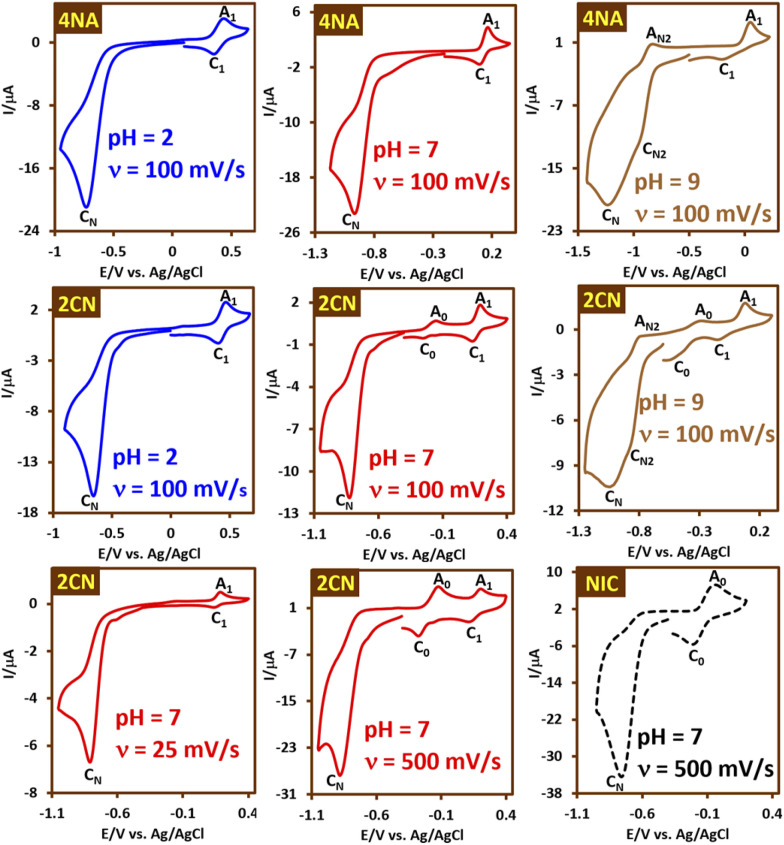
Cyclic voltammograms of 4NA (1.0 mM), 2CN (1.0 mM), and NIC (1.0 mM) at different pH values and different scan rates. Working electrode: glassy carbon electrode. Solvent: water (phosphate buffer, pH 2, *c* = 0.2 M)/ethanol (50/50 v/v). All voltammograms were recorded at room temperature.

In order to understand the effect of solution pH on the redox behavior of NIC and its metabolites, the effect of pH on the redox activity of 4NA as a model compound was first investigated (Fig. 3). As the pH increases, several changes are observed in the voltammograms, including a shift of all peaks towards more negative potentials, confirming the participation of protons in the redox process of peaks C_N_, A_1_, and C_1_. Another effect of the pH is the decrease of the peak C_1_ current with increasing pH. As shown in Scheme 2, this peak is related to the reduction of QDI to PPD. Quinonediimines (QDIs) are chemically active compounds that can participate in reactions such as hydroxylation or dimerization (reaction of QDI with PPD).^29–32^ Acidic environments suppress these reactions by protonation of PPD as well as decreasing hydroxide ion concentration, causing the peak current ratio (*I*^p^_C2_/*I*^p^_A1_) to approach unity.

**Scheme 4:**
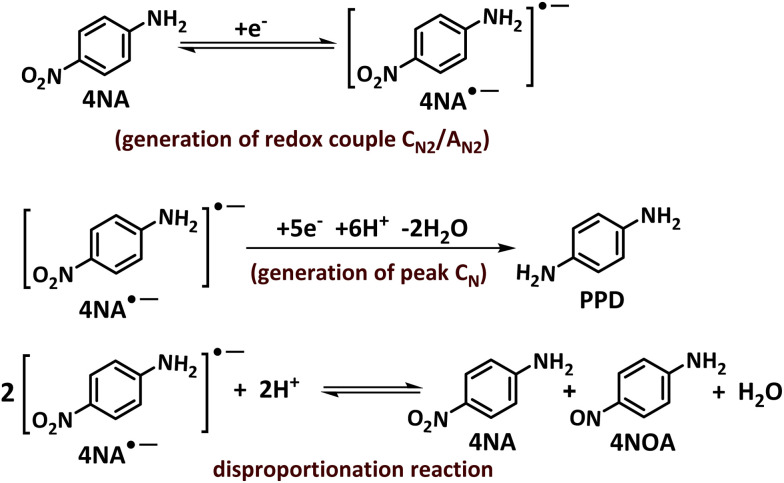
Possible proposed mechanism for the reduction of 4NA in alkaline solutions.

Conversely, as the pH of the solution increases, the rate of these reactions increases, causing the removal of QDI from the electrode surface and, consequently, the decrease of the C_1_ peak current. The third change is related to the appearance of a new cathodic peak (C_N2_) associated with the reduction of the nitro group. In alkaline solutions, a new cathodic peak (C_N2_) appears along with its anodic counterpart peak (A_N2_) in the voltammogram. In these conditions, the six-electron reduction of the nitro group occurs in two separate steps, one- and five-electron. Accordingly, the C_N2_ peak corresponds to the one-electron reversible reduction of the nitro group to its radical anion (4NA˙^−^), and the A_N2_ is its anodic counterpart. In such conditions, the C_N_ peak corresponds to the five-electron reduction of 4NA˙^−^ to PPD (Scheme 4).^33^ The presence or absence of the C_N2_ peak (and as a result, peak A_N2_) depends on the rate of the disproportionation reaction introduced in Scheme 4.

In acidic media, the rate of this reaction is high and, as a result, the radical anion produced (4NA˙^−^) is rapidly converted to 4NA and its nitroso derivative (4NOA). The result of this reaction is the elimination of the peak C_N2_. In contrast, in alkaline media, the rate of the disproportionation reaction is low and, as a result, the lifetime of the 4NA˙^−^ produced at the electrode surface is sufficient for oxidation and reduction. Under such conditions, the C_N2_ and A_N2_ peaks are observed.^33^

**Scheme 5:**
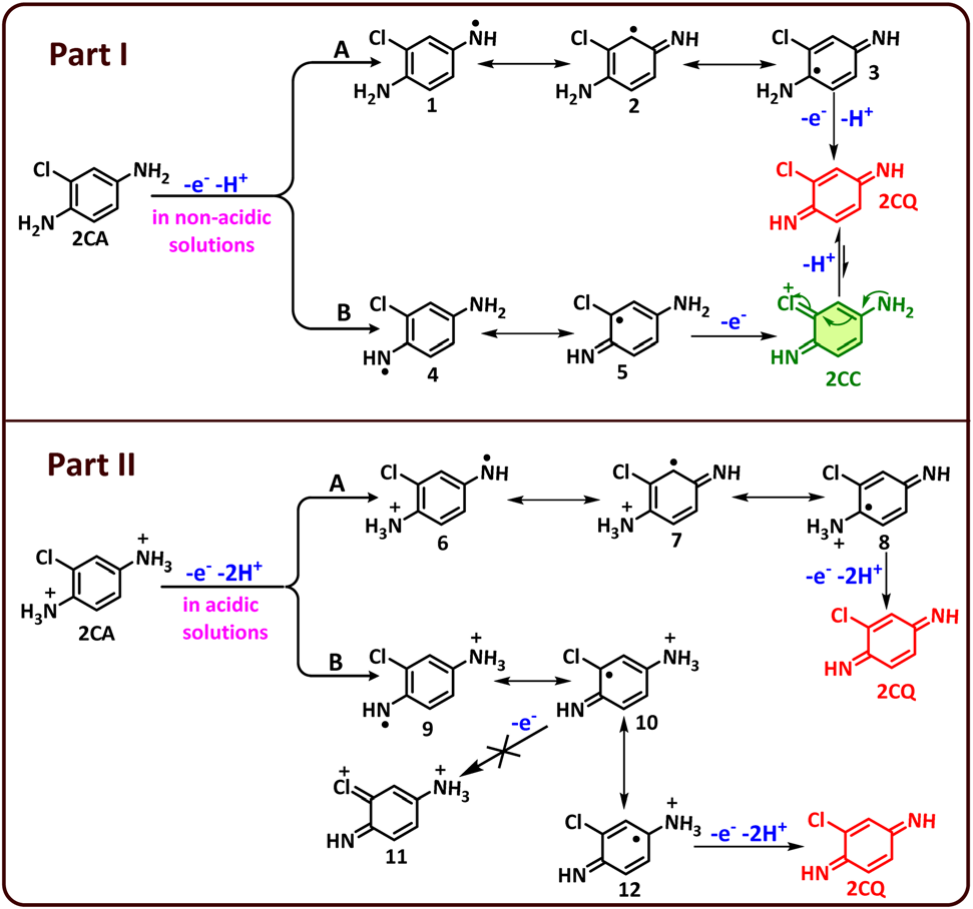
Possible proposed mechanism for the two-electron oxidation of 2CA in acidic and non-acidic solutions.

Unlike 4NA, the effect of pH on the cyclic voltammograms of 2CN is a bit more complex. Comparison of the cyclic voltammograms recorded for 2CN with 4NA shows that, in addition to the effects observed for 4NA, increasing pH causes the appearance of a new redox couple (A_0_/C_0_) (Fig. 3). Examination of the peak current ratio (*I*^p^_A0_/*I*^p^_A1_) shows that this ratio depends on the potential scan rate as well as the solution pH, so that it increases with increasing potential scan rate and/or increasing pH (Fig. 3, inset). Comparison of the structure of 2CA with that of PPD confirms that the presence of a chlorine atom in the structure of 2CA is responsible for the formation of peaks A_0_ and C_0_. Accordingly, we propose the following detailed mechanism for the two-electron oxidation of 2CA in acidic and non-acidic solutions (Scheme 5).

In the oxidation of 2CA, there are two possibilities for the first electron transfer step. This electron may be removed from the amine group at the *meta* position to the chlorine atom (pathway A) (formation of radicals 1 and 6) or from the amine group at the *ortho* position to the chlorine atom (pathway B) (formation of radicals 4 and 9). In pathway A, in both acidic and non-acidic solutions, the removal of the second electron leads to the formation of 2CQ. In pathway B and in non-acidic solutions, the first electron is removed from the amine group in the *ortho* position to the chlorine atom, and the second electron is removed from the chlorine atom, leading to the generation of the corresponding chloronium compound ((3-amino-6-iminocyclohexa-2,4-dien-1-ylidene)chloronium) (2CC).^24^ Unlike NCC (Scheme 2), 2CC is unstable and converts to its more stable form (2CQ).

**Scheme 6:**
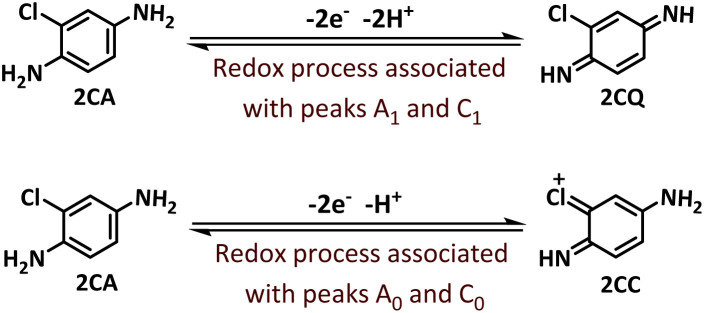
Possible proposed mechanism for the redox processes associated with peaks A_1_/C_1_ and A_0_/C_0_.

Accordingly, at low scan rates, 2CC is completely converted to 2CQ and, as a result, the A_0_ and C_0_ peaks are not observed in the voltammogram. However, at high scan rates, there is not enough time for the complete conversion of 2CC to 2CQ. Under these conditions, two anodic peaks, A_0_ and A_1_ (as well as their cathodic counterparts, C_0_ and C_1_) appear in the voltammogram (Scheme 6). Structural comparison of 2CC with 2CQ shows that 2CQ is more stable than 2CC; therefore, at low potential scan rates, 2CC converts to its more stable form, 2CQ. Similar studies were performed on NIC, and the results are shown in Scheme 2. It is recalled here that, unlike 2CC, NCC formed by oxidation of NIA is stable due to strong intramolecular electrostatic interaction. For comparison with 2CN, the cyclic voltammogram of NIC at pH 7 and a scan rate of 500 mV s^−1^ is also shown in Fig. 3. The proximity of the *E*^p^_A0_ and /*E*^p^_C0_ corresponding to 2CN (0.11 and −0.27 V, respectively) to the NIC peaks (0.02 and −0.21 V, respectively) confirms their structural similarity.

In acidic solutions, for example, at pH 2, the amine groups in the 2CA molecule are protonated (p*K*_a_ = 4.80 ± 0.10, predicted^34^). On the other hand, after oxidation, the oxidized amine is expected to be uncharged due to its lower basicity. In acidic solutions, when path B is followed, radical cations 9 and 10 are formed. Removing the second electron from the chlorine atom leads to the chloronium 11 (a dication compound) (protonated 2CC), which is a very unstable compound, and its formation seems energetically unfavorable. Accordingly, in the oxidation of 2CA in acidic environments, only peaks corresponding to the 2CA/2CQ oxidation–reduction pair (peaks A_1_/C_1_) are observed.

**Fig. 4:**
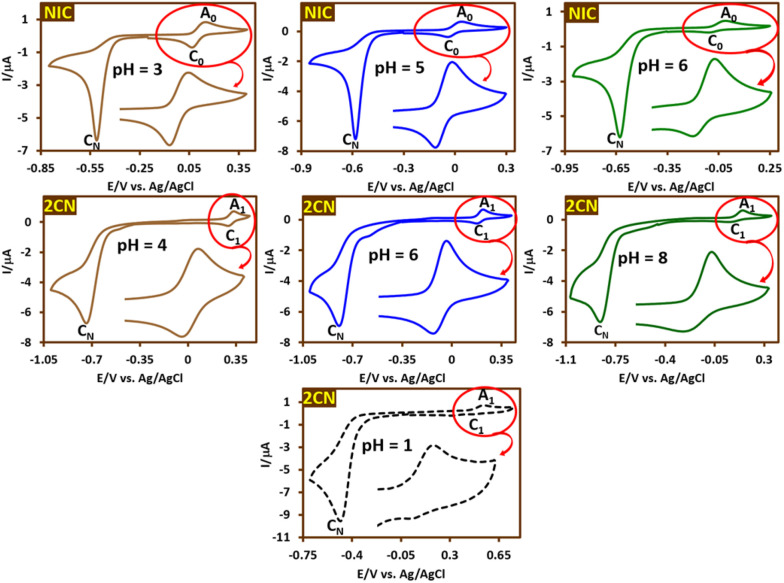
Cyclic voltammograms of NIC (1.0 mM), and 2CN (1.0 mM), at different pH solutions. Working electrode: glassy carbon electrode. Solvent: water (phosphate buffer, pH 6, *c* = 0.2 M)/ethanol (50/50 v/v). All voltammograms were recorded at room temperature.

In addition to the above, changing the pH causes a change in the peak current ratios (*I*^p^_C0_/*I*^p^_A0_, *I*^p^_C1_/*I*^p^_A1_) as well as the potential of the peaks. Fig. 4 (first row) shows the cyclic voltammograms of NIC at pH values of 3, 5, and 6. Our attention in this section focuses on the pH-dependent stability of NCC. The stability of NCC can be estimated from the peak current ratio, *I*^p^_C0_/*I*^p^_A0_. If this ratio is close to one, it means that NCC is stable on the time scale of the experiment, but if this ratio is less than one, it means that NCC is unstable (or reactive)^35^ and has participated in reactions such as hydroxylation, dimerization, *etc.*^32,35–37^ As can be seen, the cathodic peak current C_0_ (*I*^p^_C0_) decreases with increasing pH, indicating that a percentage of the produced NCCs have participated in the above reactions, especially dimerization. The rate of these reactions is pH-dependent and decreases as the pH decreases.^38^ Cyclic voltammograms of 2CN are also shown in Fig. 4 (second row). In this case, too, the reactivity of 2CQ follows the same pattern as before.

**Scheme 7:**
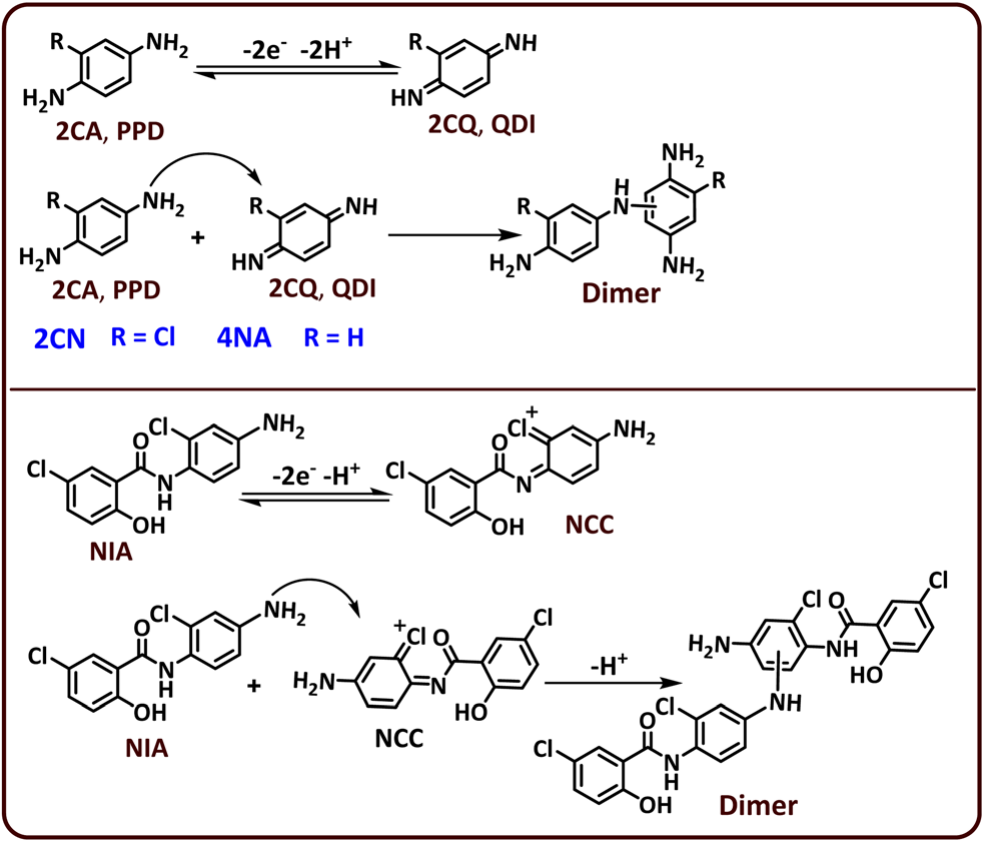
Possible proposed mechanism for the dimerization reactions.

The dimerization reaction is the coupling of NIA with NCC produced on the electrode surface (Scheme 7).

In acidic media, due to the protonation of NIA, the rate of this reaction is slow; therefore, the NCCs generated on the electrode surface are reduced during the reverse potential scan before participating in the coupling reaction.

Under these conditions, the cyclic voltammogram exhibited a Nernstian reversible behavior 
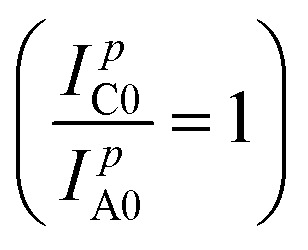
. As the pH of the solution increases, the fraction of protonated-NIAs decreases, and therefore the rate of the coupling reaction between NIA and NCC increases. Under these conditions, a percentage of the produced NCCs are involved in the coupling process, and therefore, the amount of NCC on the electrode surface decreases, which leads to a decrease in the cathodic C_0_ peak current.

Another finding regarding the pH dependence of the coupling reaction (Scheme 7) is the increase in the rate of this reaction in strongly acidic environments (*e.g.*, pH ≤ 1). Although increasing the acidity of the solution causes protonation of NIA or 2CA and decreases their nucleophilicity (slowing down the rate of the coupling reaction), if the acidity of the solution is high enough to protonate the NCC or 2CQ molecules, their electrophilicity increases and, consequently, the rate of the coupling reaction increases. Such a phenomenon is seen in the cyclic voltammogram of 2CN at pH 1. At the end of this section, it is worth noting that similar results have been obtained for 4NA.

### Potential-pH diagram

**Fig. 5:**
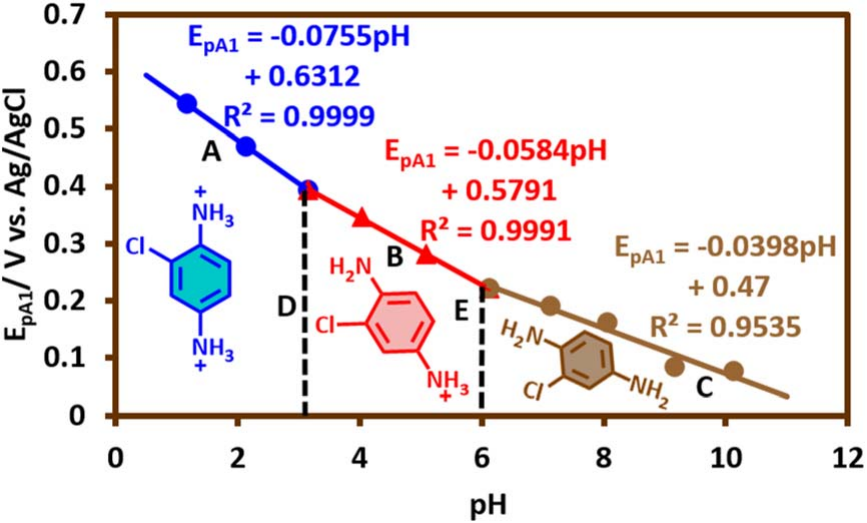
Potential-pH diagram for peak A_1_ in 2CN.

**Scheme 8:**
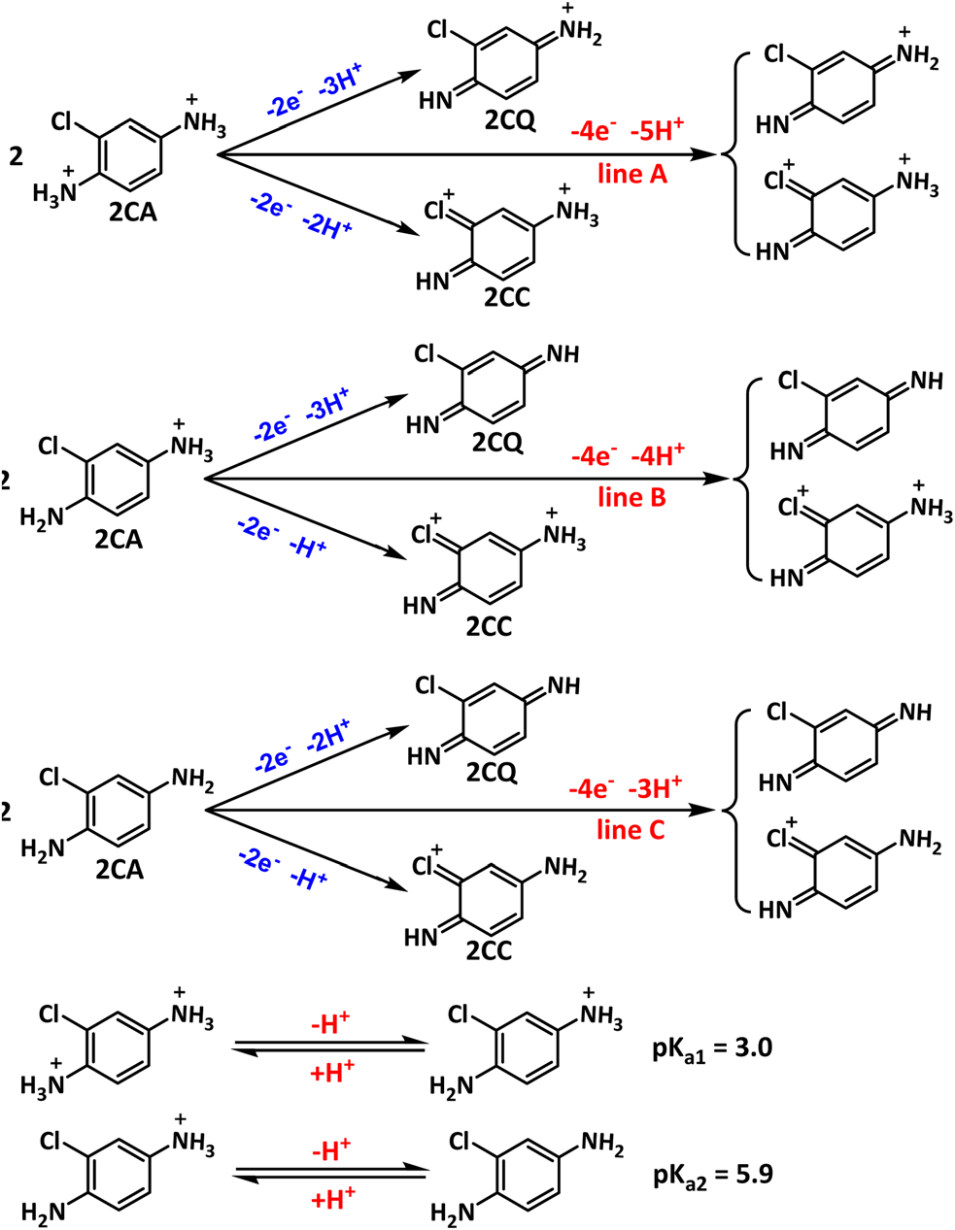
Reactions corresponding to lines A to D in the potential-pH diagram of 2CA.

In this section, the effect of pH on the peak potentials of NIC, 2CN, and 4NA is investigated. In these molecules, the potentials of all peaks shift towards less positive values with increasing pH. This result indicates that protons are involved in the redox processes associated with the peaks. The potential-pH diagram for peak A_1_ in 2CN is shown in Fig. 5. This diagram consists of three lines, A, B, and C, with different slopes. The slope of these lines is equal to the ratio of the number of protons (*m*) to the number of electrons (*n*) times 59.2 (*m*/*n* × 59.2 mV per pH). The slope of line A is 75.5 mV. Accordingly, and since the oxidation of 2CA is a two-electron process, the number of protons is calculated to be 2.5, which is definitely unacceptable and not a sign of a simple process. Accordingly, we propose the following mechanism (Scheme 8) for the oxidation of 2CA at pH values below 3. It appears that in this pH region, both amine groups in the 2CA structure are protonated. The fully-protonated-2CA is oxidized on one side by losing two electrons and three protons to the protonated-2CQ, and on the other side by losing two electrons and two protons to the protonated-2CC. Therefore, in total, two fully-protonated-2CA molecules are oxidized by losing four electrons and five protons. The slope of line A is 75.5 mV per pH, which is close to 74 mV per pH, the theoretical value for a four-electron/five-proton process.

Line B lies in the pH range between 3 and 6 and has a slope of 58.4 mV per pH. The proposed mechanism for the oxidation of 2CA in this region is also shown in Scheme 8. In this pH region, only one of the amine groups in the 2CA structure is protonated. The protonated-2CA is oxidized on the one hand by losing two electrons and three protons to 2CQ and on the other hand by losing two electrons and one proton to *protonated*-2CC. In total, in this pH range, two *protonated*-2CA molecules are oxidized by losing four electrons and four protons. The slope of line B is 58.4 mV per pH, which is close to 59.2 mV per pH, the theoretical value for a four-electron/four-proton process.

Line C is located at pH values greater than 6 and has a slope of 39.8 mV per pH. The proposed mechanism for the oxidation of 2CA in this region is also shown in Scheme 8. In this pH region, none of the amine groups are protonated. The 2CA is oxidized on the one hand by losing two electrons and two protons to 2CQ and on the other hand by losing two electrons and one proton to 2CC. In total, in pH values greater than 6, two 2CA molecules are oxidized by losing four electrons and three protons. The slope of line C is 39.8 mV per pH, which is close to 44.4 mV per pH, the theoretical value for a four-electron/three-proton process. In Fig. 5, vertical line D is the intersection of lines A and B. The acid/base equilibrium on both sides of this line is shown in Scheme 8. Accordingly, the acid constant of this equilibrium (p*K*_a_1__ of fully-protonated-2CA) was calculated to be 3.0 ± 0.03. Line E is the intersection of lines B and C. The acid/base equilibrium on both sides of this line is shown in Scheme 8. The acid constant of this equilibrium (p*K*_a_2__ of *protonated*-2CA) was calculated to be 5.9 ± 0.03.

### The effects of the potential scan rate

**Fig. 6:**
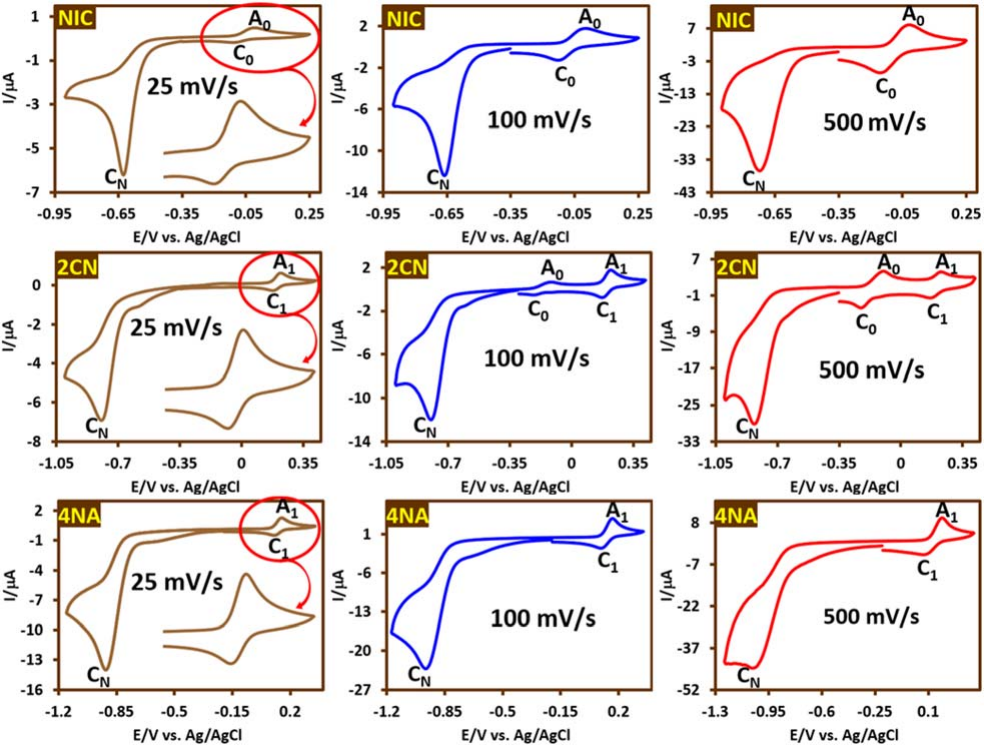
Cyclic voltammograms of NIC (1.0 mM), 2CN (1.0 mM), and 4NA (1.0 mM) at different scan rates. Working electrode: glassy carbon electrode. Solvent: water (phosphate buffer, pH 6, *c* = 0.2 M)/ethanol (50/50 v/v). All voltammograms were recorded at room temperature.

Fig. 6 shows the cyclic voltammograms of NIC at scan rates of 25, 100, and 500 mV s^−1^. As can be seen, the peak current ratio (*I*_C0_^p^/*I*_A0_^p^) at 25 mV s^−1^ is less than one, but approaches unity as the scan rate increases. This fact indicates the instability of NCC in our experimental conditions and confirms its participation in reactions such as hydroxylation or dimerization.^32,35–37^ A similar scan rate-dependent behavior is observed for 2CN, except that the peak current ratio (*I*_C1_^p^/*I*_A1_^p^) in this molecule (at a scan rate of 25 mV s^−1^) is larger than that of NIC, confirming the greater stability of 2CQ compared to NCC. Another difference is the presence of two redox peaks A_0_/C_0_ and A_1_/C_1_ in the 2CN voltammograms, which was discussed earlier. A comparison of the peak current ratios *I*_C1_^p^/*I*_A1_^p^ and *I*_C0_^p^/*I*_A0_^p^ at a scan rate of 100 mV s^−1^ shows that 2CQ is more stable than 2CC. Fig. 6 also shows the cyclic voltammograms of 4NA at scan rates of 25, 100, and 500 mV s^−1^. As can be seen, no significant differences are observed in the cyclic voltammograms of 4NA compared to 2CN.

### Reactivity toward glutathione (GSH)

**Fig. 7:**
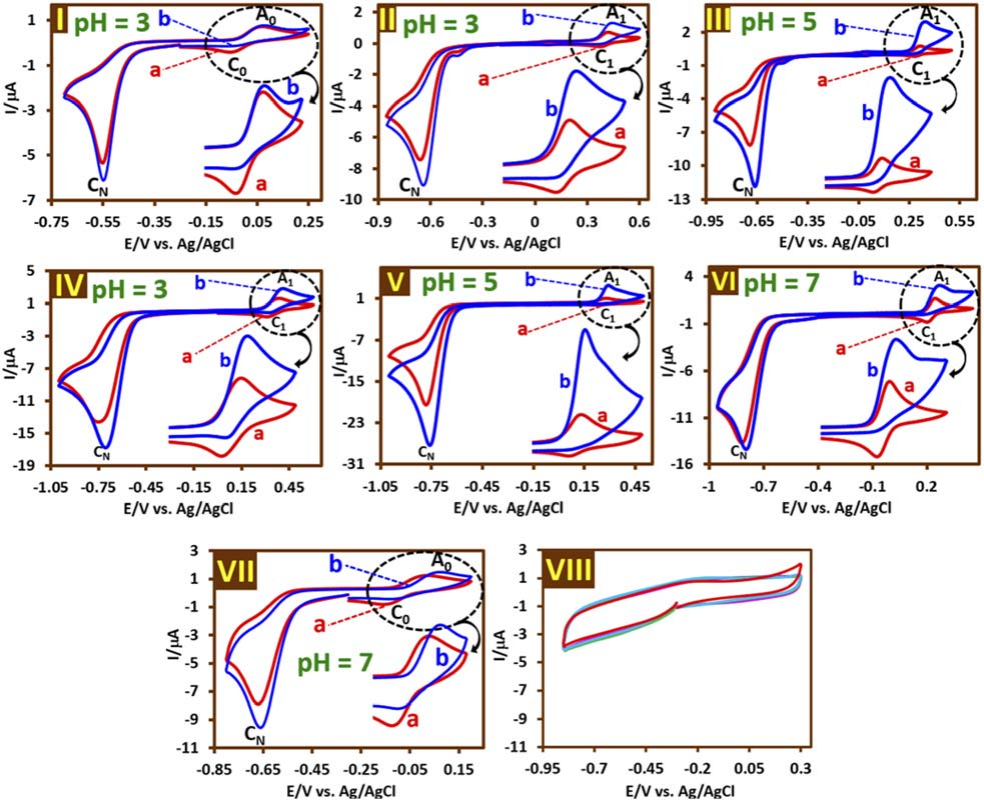
(I): cyclic voltammograms of NIC (1.0 mM) in the absence and the presence of GSH (2.0 mM). (II) and (III): cyclic voltammograms of 2CN (1.0 mM) in the absence and the presence of GSH (2.0 mM). (IV–VI): cyclic voltammograms of 4NA (1.0 mM) in the absence and the presence of GSH (2.0 mM). Scan rate: 25 mV s^−1^. (VII) cyclic voltammograms of NIC (1.0 mM) in the absence and the presence of GSH (2.0 mM) at *ν* = 100 mV s^−1^. (VIII) Cyclic voltammograms of GSH (3.0 mM) at pH values of 3.0, 5.0, 7.0, and 10.0, at *ν* = 100 mV s^−1^. Solvent: water (buffer with different pH values, *c* = 0.2 M)/ethanol (50/50 v/v). The pH values are shown on the voltammograms. Working electrode: glassy carbon electrode. All voltammograms were recorded at room temperature.

Glutathione (l-γ-glutamyl-l-cysteinylglycine) (GSH) is a tripeptide composed of three amino acids (glycine, glutamic acid, and cysteine) that is present in most mammalian tissues. Glutathione functions as a free radical scavenger, an antioxidant, and a detoxifying agent.^39^ Studies have shown that quinones,^40^ quinoneimines,^41–43^ quinonediimines,^32,42^ haloniumquinone,^44^ and chloroniumquinoneimine^24^ act as Michael acceptors and cause toxicity by forming covalent bonds with biomolecules. Glutathione has been shown to react with toxic quinoneimine produced from the oxidation of acetaminophen by enzymes such as cytochrome P-450, eliminating its toxicity.^16,17,41^ Therefore, we investigate the reaction of glutathione with toxic chloroniumquinoneimine and quinonediimines derived from NIC, 2CN, and 4NA (Fig. 7).

**Scheme 9:**
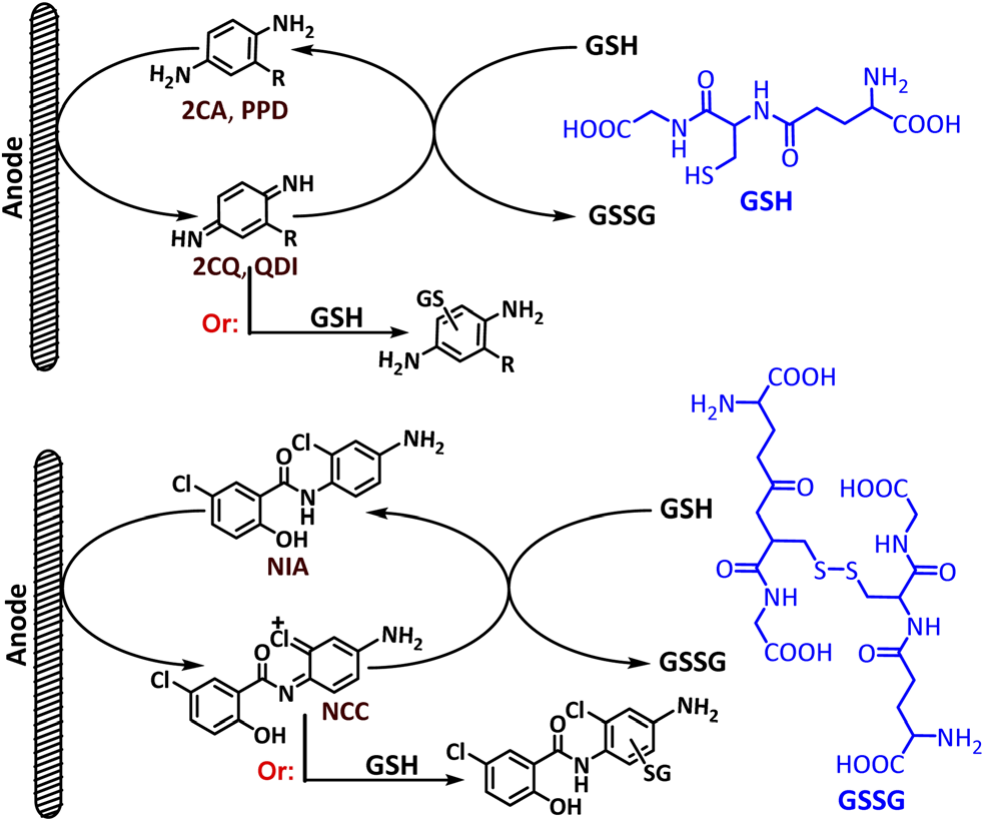
Possible proposed mechanism for the electrochemical oxidation of NIA, 2CA, and PPD in the presence of GSH.

Cyclic voltammograms *b* in Fig. 7 correspond to a solution containing one of the test compounds in the presence of GSH. The common feature of all these voltammograms, compared to the case where GSH is not present in the solution (cyclic voltammograms a), is that the cathodic peak C_1_ is eliminated or decreased, and the anodic peak A_1_ is increased. These results confirm the reaction between NCC, 2CQ, and QDI with GSH. This reaction could be a Michael addition reaction in which GSH, as a nucleophile, is added to NCC, 2CQ, or QDI*via* its sulfur atom, or an electron transfer reaction in which GSH is converted to its oxidized form (GSSG). Since in voltammetric measurements, both anodic and cathodic peak currents were perturbed in the presence of GSH (decreased cathodic peak current and increased anodic peak current), it can be concluded that the reaction between GSH and NCC, 2CQ, and QDI could also be of the electron transfer (catalytic) type (Scheme 9).

Since these experiments were performed at pH 7 (Fig. 7, part VII), which is close to the biological pH of blood (7.3–7.5),^45^ if NIC is converted to NCC in the body, it can react with important biological molecules such as proteins or nucleic acids. The rate of this reaction is proportional to the peak current ratio (*I*_A0_^p^/*I*_C0_^p^ or *I*_A1_^p^/*I*_C1_^p^), so that the larger *I*_A0_^p^/*I*_C0_^p^ (or *I*_A1_^p^/*I*_C1_^p^), the faster the reaction rate between quinonediimine (or NCC) and GSH. Accordingly, the reaction rate between 2CQ and QDI with GSH is faster than that of NCC. Since the rate of electrocatalytic reactions depends on the potential difference between the electron donor and acceptor,^46^ the closer these two potentials are, the faster the electron transfer between the two species occurs.

Since the oxidation potential of NIA (*E*_A0_^p^) (Fig. 1) is less than that of 2CA and PPD (*E*_A1_^p^), the electron transfer rate between NCC and GSH is slower than that of 2CQ and QDI, and consequently, the peak current ratio (*I*_A0_^p^/*I*_C0_^p^) of NIA in the presence of GSH is lower than *I*_A1_^p^/*I*_C1_^p^ for 2CA and PPD. Cyclic voltammograms of GSH alone at pHs 3, 5, 7, and 10 are shown in Fig. 7, part VIII. As can be seen, GSH does not exhibit significant electron transfer activity in the potential range scanned at these pHs.

**Scheme 10:**
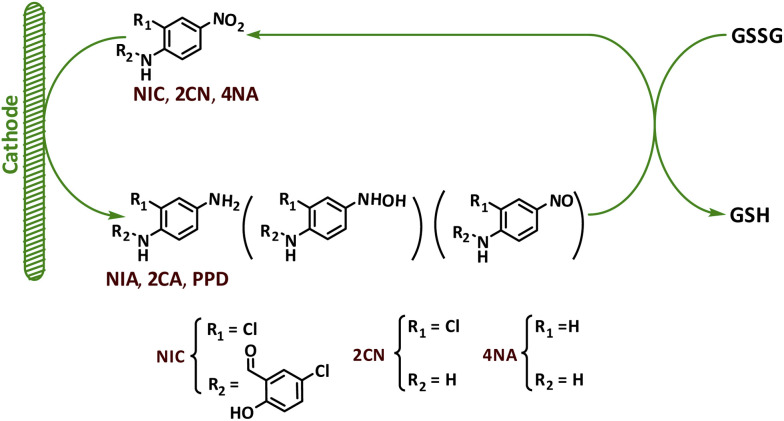
Possible proposed mechanism for the electrochemical reduction of NIA, 2CN, and 4NA in the presence of GSSG.

Another important and unique point of this study is the increase in the peak current associated with nitro reduction (C_N_) in the presence of glutathione. This phenomenon is a (catalytic) electron transfer process between the oxidized form of glutathione (GSSG) and the formed amine (or one of the formed intermediates such as nitroso or hydroxylamine), during which the amine, hydroxylamine, or nitroso is oxidized, and GSSG is reduced to GSH (Scheme 10).

The regeneration of nitro compounds at the electrode surface leads to an increase in the *I*_CN_^p^. The faster the electron transfer reaction between GSSG and the reducing species, the greater the increase in the C_N_ peak current. GSH must be converted to its oxidized form (GSSG) before this reaction can occur. It seems that the chemical oxidation of GSH to GSSG was carried out by the nitro compound. Therefore, before the voltammetry was started, GSH was oxidized to GSSG by the nitro compound (and of course, a percentage of the nitro compound was also reduced). It is worth noting that the oxidation of thiols by nitrobenzene and the formation of disulfides have been previously reported.^47^

## Conclusion

In this work, two NIC metabolites, 2CN and 5CA, were synthesized, and their redox activity was investigated along with NIC and 4NA (as a model compound). Here, we were able to fully determine the details of the redox activity of NIC. The results show that the nitro compounds NIC, 2CN, and 4NA are reduced to their amine analogs in the cathodic scan. 2CN and 4NA are then oxidized to their quinonediimine forms in the anodic scan. However, the presence of an amide group and a chlorine atom in the *ortho* position in the NIC structure has caused the oxidation of NIA to take a different route and convert to chloroniumquinoneimine (NCC). Strong intramolecular electrostatic interaction between carbonyl oxygen and positive chlorine atom stabilizes NCC. The details of the above expressions are shown in Scheme 2. The oxidation of NIC was also investigated and compared with the oxidation of 2CN and 4NA. The oxidation of these compounds is an irreversible one-electron process. NIC oxidation occurs at less positive potentials than 2CN and 4NA and, unlike 2CN and 4NA, has two overlapped anodic peaks. The presence of two oxidizable aromatic rings in the NIC structure, as well as resonance stability, are the main factors distinguishing NIC from 2CN and 4NA. The details of the above expressions are shown in Scheme 3. Other similarities and differences in the redox behavior of NIC with other metabolites mentioned are discussed in Schemes 2–8.

In this work, we also investigated the redox behavior of NIC, 2CN, and 4NA in the presence of GSH. The results show that all cathodic and anodic peaks of the mentioned compounds are perturbed in the presence of GSH. The perturbation of the A_0_ and C_0_ peaks indicates that if NIC is converted to NCC in the body by any means (*e.g.*, reduction by cytochrome P450-NADPH reductase or oxidation by cytochrome P450-catalyzed oxidation), the formed NCC can react with compounds such as amino acids, proteins, or nucleic acids. Since the reaction rate between GSH and NCC is high in test solutions with pH close to blood pH, our data predict that GSH present in the body can react with NCC and cause NIC detoxification. Another important point of this work, which has not yet been reported in literature, is the catalytic reduction of the nitro group in the presence of GSSG. The details of this reaction are shown in Scheme 10.

## Experimental section

### Apparatus, reagents, and general remarks

Niclosamide (NIC) was extracted and recrystallized with ethanol from tablets (Zagros Farmed Pharmaceutical Company, Iran). The melting point of the extracted NIC was 230 °C, consistent with the value reported in the literature.^48,49^ Furthermore, the FTIR spectrum of the extracted NIC was in good agreement with the previously reported spectrum, indicating isolation of pure NIC.^50–52^ Purification of NIC was checked by thin-layer chromatography (TLC) on silica gel using *n*-hexane/ethyl acetate (50 : 50, v/v). The materials required for the preparation of buffer solutions, including phosphoric acid, acetic acid, perchloric acid, sodium carbonate, sodium hydrogen carbonate, and sodium hydroxide, were obtained from Merck and were used without purification. Buffers were prepared in water according to recommended methods.^53^ Solvents of chloroform, acetone, acetonitrile, ethyl acetate, *n*-hexane, and ethanol were obtained from Merck and Sigma-Aldrich companies. Autolab model PGSTAT 302N Nova1.10 Niv potentiostat/galvanostat was used to perform cyclic voltammetry experiments. A three-electrode system in an undivided cell consisting of a glassy carbon (GC) disc (areas of 2.3 mm^2^) as a working electrode, an Ag/AgCl/3.0 M KCl electrode as the reference electrode, and a platinum wire as the counter electrode was used for electrochemical studies. The glassy carbon electrode was polished with alumina slurry. All electrodes were from Azar electrode, Orumieh, Iran. Aqueous solution pH was measured by a pH-meter (Metrohm/827, accuracy ± 0.1). The melting points were measured in open capillary tubes using an Electrothermal apparatus model 9100, and are uncorrected.

### Synthesis of 2-chloro-4-nitroaniline (2CN) and 5-chlorosalicylic acid (5CA)

2-Chloro-4-nitroaniline (2CN) and 5-chlorosalicylic acid (5CA) were synthesized according to the method reported by Zaazaa *et al.*^19^ The procedure was as follows: NIC (1.0 g) was dissolved in 30 mL of sodium hydroxide solution (10%) and placed in a hot water bath at 95 °C for 2.5 h. After that, the solution was cooled to room temperature and then placed on ice. The resulting precipitate, 2CN, was filtered and washed with deionized water. Purification of 2CN was carried out by thin-layer chromatography (TLC) on silica gel using *n*-hexane/ethyl acetate (60 : 40, v/v). The light-yellow powder was dried under vacuum and identified as the desired product 2CN (M.p. 110 °C, lit. 108 °C (ref. 19)). The solution after filtration contained 5CA. To this solution, concentrated HCl was added dropwise until the pH reached approximately 2, and then the resulting white precipitate, 5CA, was filtered and washed several times with distilled water. Purification of 5CA was also carried out by thin-layer chromatography (TLC) on silica gel using *n*-hexane/ethyl acetate (60 : 40, v/v) (M.p. 172–174 °C, lit. 173 °C (ref. 19)).

## Ethics approval

This article does not contain any studies with animals performed by any of the authors.

## Consent for publication

We authorize the publication of the article without any conflicts.

## Author contributions

Davood Nematollahi: supervision, project administration, resources, writing – original draft, writing – review and editing. Hadis Khazaei-Rahmati: investigation.

## Conflicts of interest

The authors declare no conflicts of interest.

## Supplementary Material

RA-OLF-D6RA02391F-s001

## Data Availability

All data generated or analyzed during this study are included in this published article. Supplementary information (SI): FTIR-spectra of NIC, 5CA and 2CN. See DOI: https://doi.org/10.1039/d6ra02391f.
