# Supplementary material for: New insight into the redox activity of niclosamide, an anthelmintic drug, and some of its metabolites. A new perspective on toxicity and detoxification
Source: RSC Adv. 2026 Jul 6. Online ahead of print. doi: 10.1039/d6ra02391f (PMC13334526; doi:10.1039/d6ra02391f)
Supplement: RA-OLF-D6RA02391F-s001 [file RA-OLF-D6RA02391F-s001.pdf]

Supplementary Information (SI) for RSC Advances.
This journal is © The Royal Society of Chemistry 2026

Supporting Information

New Insight into the Redox Activity of Niclosamide, an Anthelmintic Drug, and some of its Metabolites. A New Perspective on Toxicity and Detoxification

Davood Nematollahi,[a,b,]* Hadis khazaei-Rahmati[a]

[a]Faculty of Chemistry and Petroleum Sciences, Bu-Ali Sina University, Hamedan, Iran. Zip Code 65178-38683.

[b]Plant Chemistry Research Center, Bu-Ali Sina University, Hamedan, Iran.

*Corresponding author. Tel.: + 0098 813 8271541; Fax: +0098 813 8272404.

E-mail addresses: *nemat@basu.ac.ir, dnematollahi@yahoo.com* (D. Nematollahi).

Fax: +0098 813 8257407, Tel: +0098 813 8282807.

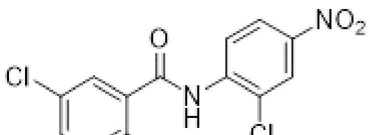

Content **Page**

Figure S1. FTIR-spectrum of niclosamide (**NIC**)………..…………………………………………………... 3

Figure S2. FTIR-spectrum of 5-chlorosalicylic acid (**5CA**) ………………………………………………. 4

Figure S3. FTIR-spectrum of 2-chloro-4-nitroaniline (**2CN**) ……………………………………….….. 5

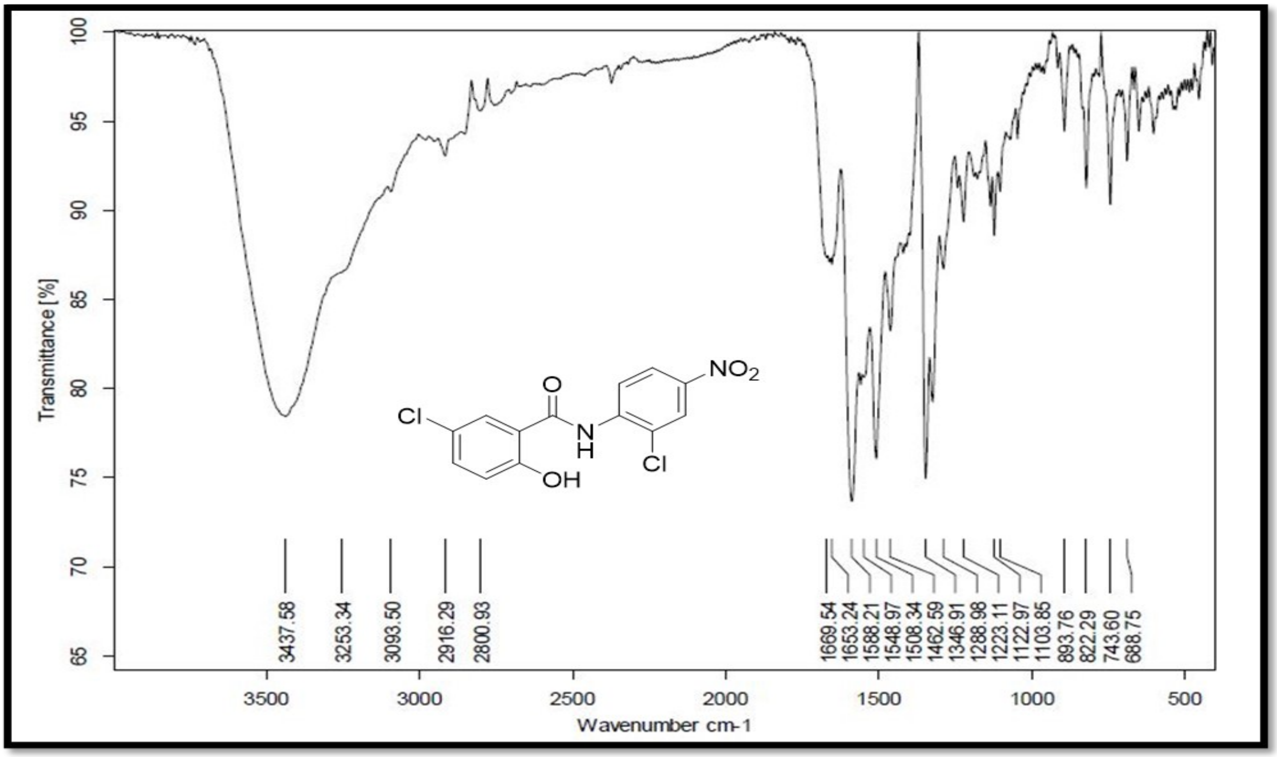

Figure S1. FTIR-spectrum of niclosamide (**NIC**).

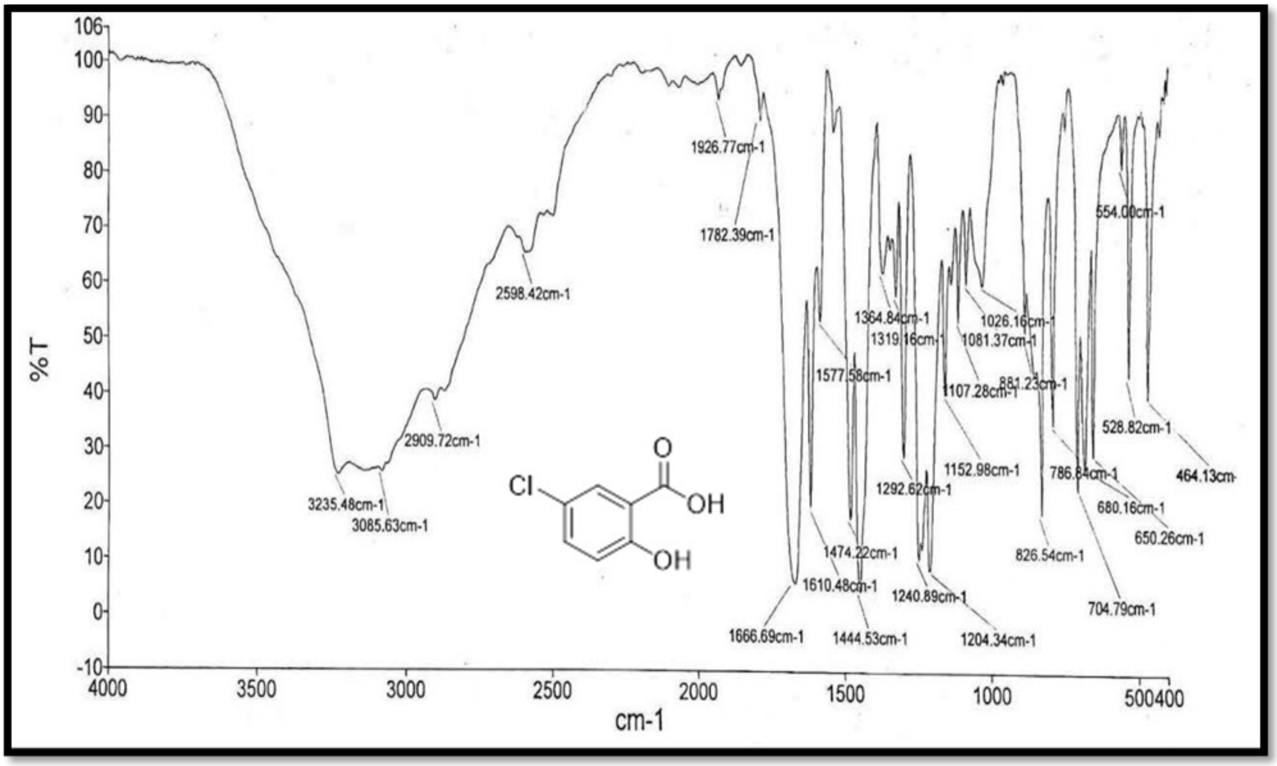

Figure S2. FTIR-spectrum of 5-chlorosalicylic acid (**5CA**).

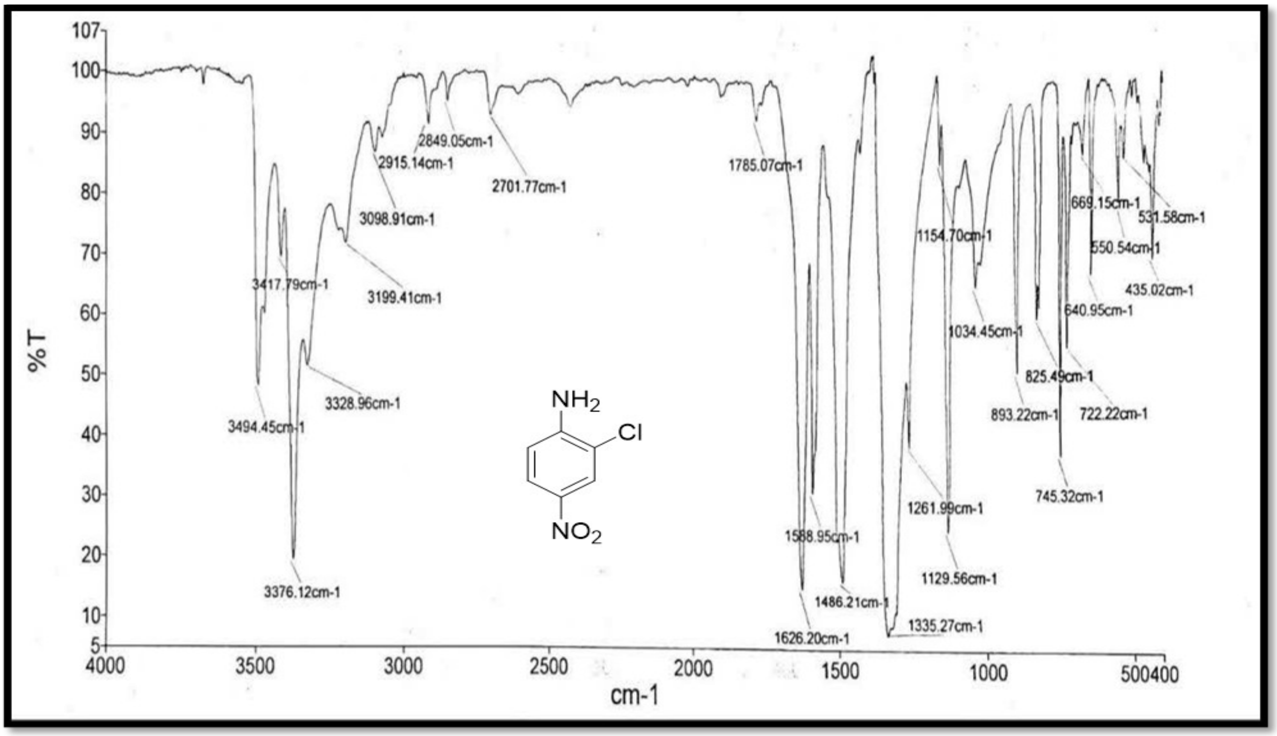

Figure S3. FTIR-spectrum of 2-chloro-4-nitroaniline (2CN).
